# A Phase 1/1B Trial of Pembrolizumab and Trametinib in Advanced NSCLC Enriched for KRAS Mutations

**DOI:** 10.1016/j.jtocrr.2025.100806

**Published:** 2025-02-12

**Authors:** Jonathan W. Riess, Matthew S. Lara, Guillaume Luxardi, Miguel Lopez de Rodas, Michiko Shimoda, Karen Kelly, Primo N. Lara, Laurel Beckett, Arta Monjazeb, Kurt A. Schalper, Emanual Maverakis, David R. Gandara

**Affiliations:** a University of California Davis Comprehensive Cancer Center, Sacramento, California; b Yale School of Medicine and Yale Cancer Center, New Haven, Connecticut

**Keywords:** NSCLC, KRAS, Immunotherapy

## Abstract

**Introduction:**

MEK inhibition (MEKi) combined with programmed death ligand 1 inhibition (immune checkpoint inhibitor [ICI]) modulates the tumor immune microenvironment. This phase 1 study evaluated sequencing schemes of MEKi and ICI with trametinib and pembrolizumab in NSCLC.

**Methods:**

In this 3+3 dose escalation study, patients with advanced NSCLC were treated with lead-in trametinib (arm A) or lead-in pembrolizumab (arm B) for cycle 1, followed by a 1.5 to 2 mg oral daily dose of trametinib (d 1–10) with pembrolizumab 200 mg intravenously every 21 days. Eligible patients with progressive disease on or after platinum-based chemotherapy were enrolled. Prior ICI was allowed. Tumor tissue was analyzed with quantitative immunofluorescence. High-parameter flow cytometry was performed on blood. Adverse events were graded using the Common Terminology Criteria for Adverse Events version 4 and efficacy was evaluated by Response Evaluation Criteria in Solid Tumors version 1.1.

**Results:**

Fifteen patients enrolled (nine arm A and six arm B) with 13 (86%) harboring *KRAS* mutations and 10 (66%) receiving prior ICI. Five patients (33%) experienced at least one grade greater than or equal to 3 treatment-related adverse event including one dose-limiting toxicity (grade 3 esophagitis). Two patients had a partial response (ORR = 14%). Trametinib lead-in was associated with decreased T-regulatory cells and myeloid-derived suppressor cells (*p* = 0.002 and *p* = 0.05, respectively).

**Conclusions:**

The activity of trametinib and pembrolizumab is modest in NSCLC with increased toxicity compared with programmed death ligand 1 blockade alone. The recommended phase 2 dose for the combination is 2 mg of oral trametinib (d 1–10) and 200 mg of intravenous pembrolizumab every 21 days, with lead-in trametinib. Adverse events were comparable with other MEKi and ICI combination studies. Though limited clinical activity was observed, lead-in MEKi may induce favorable immune cell alterations.

## Introduction

Lung cancer is the leading cause of cancer-related death in the United States, estimated to account for over 125,000 deaths in 2024.^1^ NSCLC predominates and consists of adenocarcinoma, squamous cell carcinoma, and other less common histologies. Prominent progress has been made in the identification and therapeutic targeting of unique genetic or molecular alterations of lung adenocarcinomas, including activating mutations in oncogenes such as *EGFR* and *KRAS* mutations.^2^^,^^3^

The *KRAS* oncogene is mutated in approximately 25% of lung adenocarcinomas and the RAS–RAF–MAPK pathway is frequently activated or dysregulated in NSCLC.^4^ Engagement and activation of the MAPK signaling pathway ultimately results in increased tumor cell proliferation and survival, reduced apoptosis, and increased tumor metastasis.^5^ About half of *KRAS* mutations in NSCLC are G12C amino acid substitutions where the direct KRAS G12C inhibitors adagrasib and sotorasib are approved.^6^^,^^7^ There are currently no approved targeted therapies for KRAS non-G12C amino acid substitutions, but several pan-RAS and KRAS G12D inhibitors are at various stages of clinical development.^8^

MEK inhibition (MEKi) alone has insufficient clinical activity in *KRAS* mutant NSCLC, but preclinical evidence suggests that MEK inhibitors may potentiate immune checkpoint inhibitor (ICI) therapy.^5^^,^^9^ For example, in a murine colorectal *KRAS* mutant cancer model, MEKi decreased naive T-cell priming but increased antigen-specific CD8+ T-cells by protecting them from death associated with chronic T-cell receptor stimulation.^10^ The decrease in CD8+ T-cell death contributed to the intratumoral accumulation of antigen-specific effector CD8+ T-cells with capacity for tumor elimination.^10^ Nevertheless, MEKi may also up-regulate programmed cell death protein-1 (PD-1) expression on effector T-cells, which could mark both activation and/or suppression of cancer-specific T-cell responses. As one example, in *KRAS* mutant NSCLC, selumetinib therapy was associated with higher PD-1 expression on CD8+ T-cells. But this same therapy also decreased the frequency of T-regulatory cells (Tregs).^11^ The latter observation might be beneficial to someone receiving ICIs.^12^ Together these findings provide the rationale for designing MEKi-ICI combination strategies for the treatment of *KRAS* mutant NSCLC.

The timing of MEKi with respect to ICIs may also be of importance to maximize clinical impact. In a syngeneic colorectal *KRAS* mutant cancer model, tumor inhibition was superior when engrafted mice were first treated with MEKi followed by the addition of ICIs. This strategy also resulted in increased tumor-infiltrating CD8+ T-cells and a decrease in tumor growth.^13^

Intermittent versus continuous dosing schemes of MEKi and ICIs in combination also impact the immune response and antitumor activity of the regimen. Continuous exposure to MEKi may lead to resistance through feedback reactivation of the ERK pathway.^14^^,^^15^ Intermittent dosing may better maintain T-cell activation and proliferation by preventing feedback activation of the ERK pathway, thereby enhancing immunologic and antitumor activity.^15^, ^16^, ^17^ In the KEYNOTE-022 clinical trial that combined the MEKi trametinib with the PD-1 antibody pembrolizumab, higher response rates were noted with intermittent dosing compared with a concurrent continuous dosing schedule of trametinib.^18^

Given the preclinical and clinical evidence of differences in the tumor immune microenvironment and clinical activity with lead-in and intermittent doses of MEKi with PD-1 blockade in solid tumors, this study was initiated to determine safety, tolerability, and preliminary efficacy of intermittent MEKi with trametinib in combination with pembrolizumab, comparing lead-in trametinib versus lead-in pembrolizumab in NSCLC with an emphasis on *KRAS* mutant lung cancer.

## Materials and Methods

### Eligibility

The study was an open-label phase 1 trial conducted at the UC Davis Comprehensive Cancer Center between 2018 and 2021. Eligible patients were more than or equal to 18 years old with histologically or cytologically confirmed stage IV NSCLC with progression on platinum-based chemotherapy, Eastern Cooperative Oncology Group performance status of 0 or 1, and an anticipated life expectancy of more than or equal to 6 months. Prior PD(L)1 inhibitor treatment was allowed but not mandated for dose escalation. Patients were required to have adequate end-organ function. Treated and controlled brain metastases were allowed. The planned dose expansion phase included only patients with NSCLC harboring a *KRAS* mutation and without previous exposure to PD(L)1 inhibitors. Patients with a history of autoimmune disease or pneumonitis were not allowed.

The UC Davis Institutional Review Board approved the study. All patients were required to provide written informed consent before participating, and all procedures were undertaken in accordance with the Declaration of Helsinki.

### Study Design and Treatment

The study schema is summarized in Supplementary Figure 1. This is a phase 1/1B trial that tested trametinib and pembrolizumab in patients with advanced NSCLC. The cycle length was 3 weeks. There were two treatment arms: arm A (n = 12 maximum planned patients) which had a lead-in of trametinib for one cycle before proceeding to the combination with pembrolizumab; and arm B (n = 12 maximum planned patients) which had a lead-in of pembrolizumab for one cycle before proceeding to the combination with trametinib. In the phase 1 component, a limited dose exploration 3+3 scheme was employed (Supplementary Table 1) that had a starting dose level (DL1) consisting of trametinib (1.5 mg orally, d 1–10) and pembrolizumab (200 mg intravenous). Subsequent patient cohorts were either advanced to the higher dose level (DL2) or a lower dose level (DL1), dependent on the frequency and nature of dose-limiting toxicities (DLTs) seen in DL1.

### End Points and Statistical Design

This study sought to define the DLT, maximum tolerated dose (MTD), and recommended phase 2 dose (RP2D) for the combination of trametinib and pembrolizumab in patients with advanced NSCLC.

All toxicities were graded using the National Cancer Institute Common Terminology Criteria for Adverse Events version 4.0. The occurrence of any grade 3 (G3) or higher toxicities during the first two cycles (i.e., after completion of the lead-in cycle and the first cycle where both pembrolizumab and trametinib are administered) were considered a DLT, if judged to be possibly, probably, or definitely related to study drug administration. The goal of the dose escalation portion was to assess the safety of the combination after pembrolizumab or trametinib lead-in; therefore, patients must have completed the lead-in phase and have received both trametinib and pembrolizumab concurrently to be evaluated for a DLT. A DLT-assessable subject was defined as a subject who received treatment for the 6-week DLT observation period and who received at least 80% of all planned treatments.

An event was considered a DLT if it occurred during the first two cycles of treatment (first 6 weeks after initiating treatment) and met at least one of the following criteria: (1) clinically significant hematologic toxicity; (2) clinically significant G3 or higher nonhematologic toxicity that has not been previously identified for either pembrolizumab or trametinib and cannot be controlled with routine supportive measures; (3) clinically significant G3 or higher nonhematologic toxicities that are known to occur with either pembrolizumab or trametinib but that cannot be controlled using the recommended supportive measures; (4) drug-related toxicity, regardless of the Common Terminology Criteria for Adverse Events grade, that results in an interruption of any component of study therapy during cycle 1 for more than 21 consecutive days and cannot be controlled within 2 weeks from its onset; (5) any other grade 2 or greater nonhematological toxicity that in the judgment of the investigator is dose limiting, with the exception of mild or moderate immune-mediated adverse reactions or symptomatic endocrinopathy attributable to pembrolizumab; and (6) for liver function tests, alanine transaminase (ALT) or aspartate transaminase (AST) greater than or equal to eight times upper limit of normal (ULN), ALT or AST greater than or equal to five times ULN but less than eight times ULN, persisting for 2 weeks or longer, or ALT or AST three times ULN if associated with the appearance or worsening of symptoms of hepatitis. Clinically insignificant laboratory values of any grade were not considered dose-limiting.

Dose escalation proceeded within each cohort according to the scheme summarized in Supplementary Table 1. MTD was defined as the highest dose tested in which one or fewer out of six patients experienced a DLT. The RP2D was the dose level and sequence deemed by the study team to be the most appropriate for advancing into the next stage of investigation on the basis of the subsequent DLTs, MTD, and any preliminary evidence of efficacy that favored one sequence or dose level over the other.

### Assessments

Baseline imaging included computed tomography of the chest, abdomen, and pelvis and magnetic resonance imaging or computed tomography–based brain imaging. After baseline imaging, patients were assessed for response using the same modality every two cycles (∼6 wk). Patients were followed after completion of protocol-based therapy or until death, whichever occurred first. Response was evaluated using the Response Evaluation Criteria in Solid Tumors version 1.1.

### Tissue PD-L1 Immunohistochemistry and Multiplexed Immunofluorescence Staining

To explore the molecular mechanisms underlying any signs of early efficacy, quantitative immunofluorescence on baseline archival tumor specimens obtained within 6 months of study enrollment or fresh biopsy before treatment was performed to determine the levels of major tumor-infiltrating lymphocytes (TILs) subpopulations including CD4+ helper T-cells, CD8+ effector T-cells, and CD20+ B-cells. Multiplexed TILs immunofluorescence staining analytical validation and quantification are described in previous publications.^19^, ^20^, ^21^ Whole-tissue section slides were deparaffinized and subjected to antigen retrieval using ethylenediaminetetraacetic acid buffer (Sigma-Aldrich) at a pH of 8.0 and boiled for one hour at 96°C in a pressure-boiling container (PT module, Lab Vision). Slides were then incubated with dual endogenous peroxidase block (#S2003: Dako) for 10 minutes at room temperature. Nonspecific antigens were blocked by a 30-minute incubation in 0.3% bovine serum albumin in Tris-buffered saline with Tween. The sequential multiplexed immunofluorescence protocol was performed using isotype-specific primary antibodies to epithelial tumor cells (Cytokeratin Alexa-488 conjugated, clone EA1/EA3, eBioscience), helper T-cells (CD4 IgG, 1:100, clone SP35, SpringBio), cytotoxic T-cells (CD8 IgG1k, 1:250, clone C8/144B, Dako), and B-cells (CD20 IgG2a, 1:150, clone L26, Dako). Secondary antibodies and reagents used were anti-rabbit Envision (K4003, Dako), with biotinylated tyramide or Streptavidine-Alexa750 conjugate (PerkinElmer), anti-mouse IgG1k antibody (1:100, eBioscience) with Cy3-tyramide (PerkinElmer) and anti-mouse IgG2a antibody (1:200, Abcam) with Cy5-tyramide (PerkinElmer). Nuclei were highlighted using 4’,6-diamidino-2-phenylindole. The protocol included the elimination of residual horseradish peroxidase activity between incubations with secondary antibodies by exposing the slides twice for seven minutes to a solution containing benzoic hydrazide (0.136 mg) and hydrogen peroxide (50 μl). Finally, slides were mounted with ProlongGold.

The levels of immune cell markers stained with multiplexed immunofluorescence were scored using the AQUA method of automated quantitative immunofluorescence using spatial molecular compartments and co-localization strategies.^19^, ^20^, ^21^ PD-L1 protein expression was evaluated using conventional chromogenic immunohistochemistry using a clinical-grade assay (22C3) and scored using light microscopy by a pathologist in a semiquantitative fashion using the tumor proportion score.

### Flow Cytometry on Peripheral Blood

To assess the systemic immunologic effects of the pembrolizumab and trametinib combination therapy, blood was collected at serial time points: at baseline before cycle 1 (−3 d), before cycle 4 (−3 d), and before cycle 7 (−3 d). Peripheral blood mononuclear cells were then isolated and analyzed by high-parameter flow cytometry per previously described methods.^22^^,^^23^ Briefly, peripheral blood mononuclear cells were thawed and incubated with Fc-block (BD Bioscience, Franklin Lakes, NJ) on ice for 15 minutes. Then, cells were stained with specific antibody cocktails (Supplementary Table 2) on ice for one hour and then stained with a LIVE or DEAD fixable green dead cell stain kit (Invitrogen, Carlsbad, CA) for 30 minutes at room temperature. Cells were washed after each step using phosphate buffered saline containing 0.5% bovine serum albumin and before being processed on a BD Fortessa flow cytometer (BD Bioscience, Franklin Lakes, NJ). Data were analyzed using FlowJo software version 10.6.2 (Tree Star Inc. Ashland, OR). Post hoc comparisons were stratified by patients with a partial response (PR) to treatment or stable disease (SD) versus those with progressive disease (PD).^24^ The receiver operator characteristic curve (ROC) was constructed and the area under the curves (AUCs) was calculated for immune cell populations of interest and outcomes of clinical benefit.

## Results

### Patients

Patient characteristics are summarized in Table 1. The median age was 69 years (32–81 y). There were six male (40%) and nine female (60%) participants. All patients had lung adenocarcinoma with 13 patients (86%) having *KRAS* mutations (three G12C and 10 non-G12C). Meanwhile, one patient (7%) had BRAF non-V600E and one (7%) lacked detectable oncogenic driver mutations. Ten patients (67%) had a history of smoking. Ten patients (66%) had negative PD-L1 expression, three (20%) had low to moderate (1%–49%) PD-L1, and one patient (7%) had high (50%) PD-L1. All participants had prior platinum-based chemotherapy and 10 patients (66%) had prior ICI.

**Table 1:** Summary of Patient Characteristics

Characteristics	Value
Median age (range)	69 y (32–81 y)
Gender, n (%)	
Male	6 (40)
Female	9 (60)
Mutation status, n (%)	
KRAS G12C	3 (20)
KRAS non-G12C	10 (66)
BRAF non-V600E	1 (7)
RAS wild type	1 (7)
Smoking status, n (%)	
Current	0 (0)
Former	10 (67)
Never	5 (33)
PD-L1 expression (TPS 22C3), n (%)	
0%	10 (66)
1%–49%	3 (20)
50%	1 (7)
Not available	1 (7)
Treatment, n (%)	
Prior chemotherapy	15 (100)
Prior immunotherapy	10 (66)

TPS, tumor proportion score.

### Safety

Accrual to dose escalation alternated between lead-in trametinib (arm A) and lead-in Pembrolizumab (arm B). Three patients were enrolled in DL1 in arm A (trametinib lead-in). No DLTs were observed. Three patients were enrolled at DL1 in arm B (pembrolizumab lead-in). No DLTs were observed in this cohort. Three more patients were then enrolled at DL2 in arm A with one DLT (G3 esophagitis) observed. Three patients were then enrolled at DL2 arm B with no DLTs observed. A G3 pneumonitis event was also observed in arm B at DL2, which occurred outside the protocol-specified DLT period. This patient eventually developed grade 5 respiratory failure after being placed on comfort care in the setting of decompensation from acute pulmonary emboli and multimicrobial bacterial and viral (coronavirus) pneumonia. Additional potential G3 treatment-related adverse events (TRAEs) of note include: anemia (DL2 arm A), retinal detachment (DL1 arm A), and diarrhea (DL1 arm A, DL2 arm A). The toxicity data for this trial are summarized in Table 2.

**Table 2:** Treatment-Related Adverse Events, All Grades, by Dose Level

Adverse Event/Grade	Dose Level 1, Cohort A (n = 3)		Dose Level 1, Cohort B (n = 3)		Dose Level 2, Cohort A (n = 6)		Dose Level 2, Cohort B (n = 3)	
	1 or 2	3+	1 or 2	3 +	1 or 2	3+	1 or 2	3+
Abdominal pain	0	0	0	0	2	0	0	0
Alanine aminotransferase increased	0	0	0	0	2	0	0	0
Alkaline phosphatase increased	0	0	1	0	2	0	0	0
Anemia	0	0	0	0	0	1	0	0
Anorexia	2	0	1	0	0	0	0	0
Arthralgia	1	0	0	0	0	0	0	0
Aspartate aminotransferase increased	0	0	0	0	3	0	1	0
Cheilitis	0	0	0	0	1	0	0	0
Chills	0	0	0	0	1	0	0	0
Constipation	1	0	0	0	2	0	1	0
CPK increased	2	0	0	0	1	0	0	0
Dehydration	0	0	0	0	0	0	1	0
Diarrhea	1	1	1	0	4	1	2	0
Dizziness	0	0	0	0	0	0	1	0
Dry skin	0	0	0	0	0	0	1	0
Dyspnea	0	0	0	0	2	0	1	0
Esophagitis	0	0	0	0	0	1	0	0
Fatigue	2	0	0	0	3	0	2	0
Fever	0	0	0	0	1	0	0	0
Gastroesophageal reflux disease	1	0	0	0	0	0	0	0
Generalized muscle weakness	0	0	0	0	0	0	1	0
Headache	1	0	0	0	0	0	0	0
Hyperglycemia	0	0	0	0	1	0	0	0
Hypertension	0	0	0	0	1	0	0	0
Hypoalbuminemia	1	0	0	0	1	0	1	0
Hyponatremia	2	0	0	0	1	0	0	0
LDH increased	0	0	0	0	0	0	1	0
Malaise	0	0	0	0	0	0	1	0
Myalgia	1	0	0	0	0	0	0	0
Nausea	1	0	2	0	3	0	1	0
Neutrophil count decreased	0	0	0	0	1	0	0	0
Oral hemorrhage	0	0	0	0	1	0	0	0
Oral pain	0	0	0	0	1	0	0	0
Platelet count decreased	0	0	1	0	0	0	0	0
Pneumonitis	0	0	0	0	0	0	0	1
Pruritus	1	0	1	0	1	0	2	0
Rash acneiform	2	0	2	0	1	0	1	0
Rash maculo-papular	2	0	0	0	4	0	2	0
Respiratory Failure	0	0	0	0	0	0	0	1^a^
Retinal detachment	0	1	0	0	0	0	0	0
Serum amylase increased	0	0	0	0	1	0	0	0
Vomiting	0	0	0	0	2	0	0	0
Wheezing	0	0	0	0	1	0	0	0

Dose level 1:1.5 mg trametinib PO three times per week, 200 mg pembrolizumab IV once every three weeks.

Dose level 2:2 mg trametinib PO three times per week, 200 mg pembrolizumab IV three times per week.

Cohort A: lead-in of trametinib for one cycle before proceeding to the combination with pembrolizumab.

Cohort B: lead-in of pembrolizumab for one cycle before proceeding to the combination with trametinib.

CPK, creatine phosphokinase; IV, intravenous; LDH, lactate dehydrogenase; PO, per orally.

a Grade 5 event.

### Efficacy

**Figure 1:**
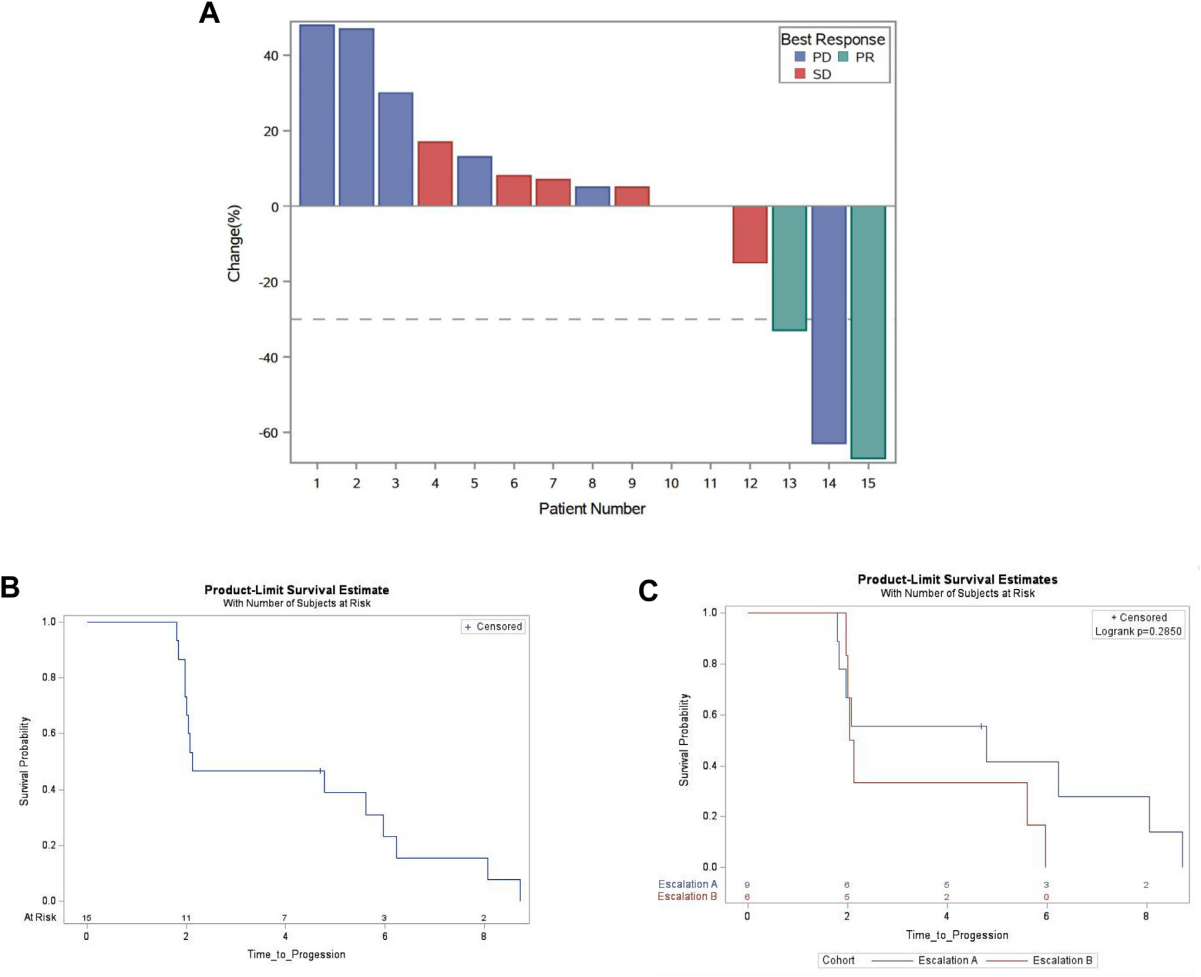
Antitumor activity of trametinib and pembrolizumab in advanced NSCLC in the study. *(A)* Waterfall plot of best response. *(B)* Kaplan-Meier curve of PFS in the evaluable population (median PFS = 2 mo, 95% CI: 2–5.5 mo in the overall population) and *(C)* by cohort with blue as Cohort A (lead-in trametinib) and red as Cohort B (lead-in pembrolizumab). CI, confidence interval; PFS, progression-free survival.

Two PRs (2 of 15, 13%) were noted (both in patients with *KRAS* G12C mutations: one patient with prior de novo progression on nivolumab and in one patient who was PD-1 inhibitor naive) (Fig. 1*A*). Six patients had SD as the best response and seven patients had PD as the best response (Table 3). The median progression-free survival (median progression-free survival [mPFS]) was 2.13 months (95% confidence interval [CI]: 2–6 mo) with no clear difference between arm A (lead-in trametinib) (mPFS 4.79 mo, 95% CI: 1.8–8.1 mo) and arm B (lead-in pembrolizumab) (mPFS = 2.08 mo, 95% CI: 1.97–5.97 mo), albeit limited by the small sample size in each arm (Fig. 1*B* and *C*).

**Table 3:** Summary of Key Clinical, Pathologic, and Molecular Characteristics and Outcomes of the Study Patients

Pt ID	Gender	Age (y)	Arm	Dose Level	KRAS Status	PD-L1 Expression	Prior PD(L)1 Treatment	Best Response	PFS (mo)
UCD-259-001	F	69	A	1	*KRAS* Wt,	0	Yes	SD	8.9
UCD-259-002	F	74	A	1	*KRAS* G12C	ND	Yes	PR	6.3
UCD-259-003	M	67	A	1	*KRAS* G12D	0	No	SD	8.2
UCD-259-004	M	32	B	1	*KRAS* G12V	0	No	PD	2.1
UCD-259-005	F	72	B	1	*KRAS G12C*	0	Yes	PD	2.2
UCD-259-006	M	63	B	1	*KRAS* Q61H	0	Yes	PD	2
UCD-259-007	M	58	A	2	*KRAS* G12V	40	Yes	SD	4.9
UCD-259-008	F	72	A	2	*KRAS* G12A	0	Yes	PD	2
UCD-259-009	N/A	52	A	2	*KRAS* G12D	0	Yes	SD	>2.1^a^
UCD-259-010	F	71	B	2	*KRAS* Q61L	100	Yes	SD	6.1
UCD-259-011	F	69	B	2	*KRAS* G12C	0	No	PR	5.7
UCD-259-013	M	81	B	2	*BRAF* G469A	0	No	PD	2
UCD-259-014	F	69	A	2	*KRAS* G12D	0	No	PD	1.9
UCD-259-015	F	49	A	2	*KRAS* G12D	20	Yes	SD	>4^a^
UCD-259-016	M	39	A	2	*KRAS* G12V	1	Yes	PD	1.8

CI, confidence interval; F, female; M, male; PD, progressive disease; PD-1, programmed cell death protein-1; PD-L1, programmed death-ligand 1; PFS, progression-free survival; Pt, patient; PR, partial response; SD, stable disease.

a Censored for the date of the last contact.

### Immune Correlative Studies

**Tissue Analysis of TILs.** Twelve of the 15 patients had tissue assessable at baseline (80%). Several notable trends were observed with clinicopathological variables showing differences in immune cell types and compartment location. There was a trend of higher levels of TILs in older patients (>65 y). The amount of T- and B-cells was higher in female individuals than in male individuals (*p* = 0.02). The tumor compartment in nonsmokers displayed higher levels of CD8+ T-cells (*p* = 0.03). Patients previously treated with immunotherapy reported a trend toward higher T-cell infiltration than those treated only with chemotherapy. Results of TIL measurements representing CD4+, CD8+, and CD20+ cells by treatment arm and patient demographics are included in Supplementary Table 3 and Supplementary Figure 2.

**Figure 2:**
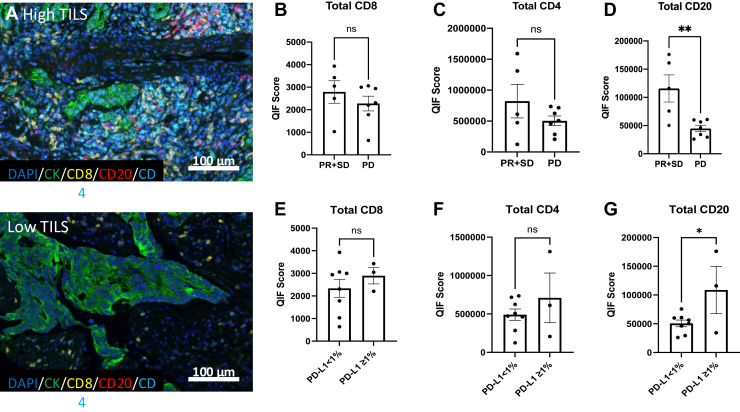
QIF detects baseline TILs associated with antitumor activity and PD-L1 expression in patients treated with trametinib and pembrolizumab. *(A)* Representative QIF panels of high (top) and low (bottom) TILs. CD8+, CD4+, and B-Cells associated with clinical activity (PR + SD versus PD) *(B–D)* and PD-L1 expression *(E–G)*. PD, progressive disease; PD-L1, programmed death-ligand 1; PR, partial response; QIF, quantitative immunofluorescence; SD, stable disease; TIL, tumor-infiltrating lymphocyte. ∗ p<0.05, ∗∗ p<0.01.

The levels of TILs were comparable across trial arms and dose levels at baseline. Tumor specimens with higher baseline TILs displayed a higher level of antitumor activity: CD8+ and CD4+ T-cells were numerically higher in patients who had evidence of antitumor activity (PR or SD) and CD20+ B-cells were significantly higher in patients who had disease control (PR + SD) compared with patients who had PD (Fig. 2*A*, *B*, *C*, *D*). PD-L1 positive tumors (tumor proportion score > 1%) displayed higher T- and B-cell levels than did PD-L1 negative tumors, with CD20+ B-lymphocytes demonstrating statistical significance (Fig. 2*E*, *F*, *G*).

**Figure 3:**
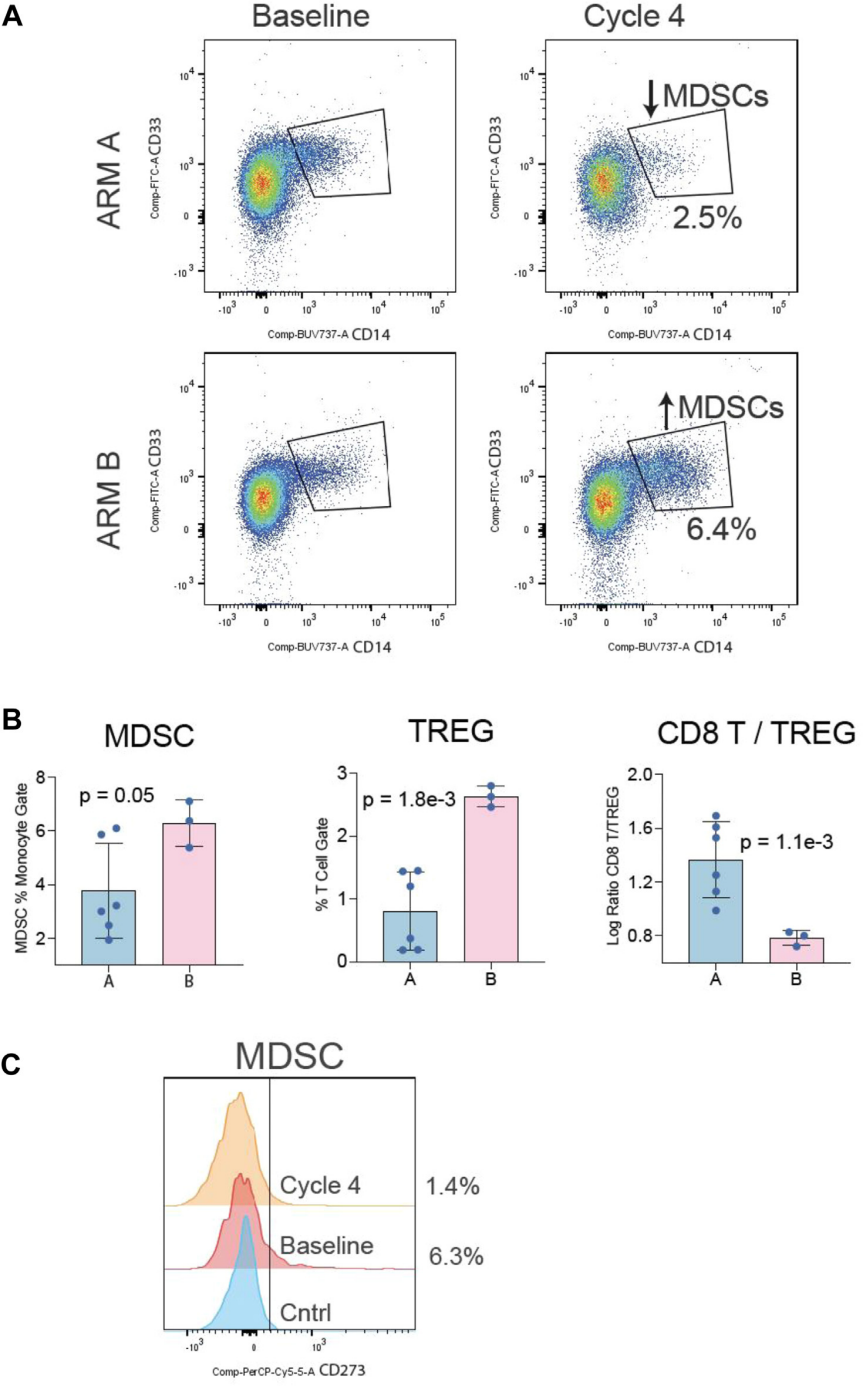
Flow cytometric analysis of immune cell populations with and without MEKi lead-in. *(A)* Flow cytometric analysis reveals that the frequency of MDSCs is increased in arm B (no MEKi lead-in). *(B)* Bar graphs of different immune cell populations in arm A (MEKi) versus arm B (no MEKi). Error bars indicating seventy-fifth and twenty-fifth percentiles. Arm A was associated with fewer MDSCs and Tregs. Arm A was also associated with a lower frequency of MDSCs, Tregs, and a higher ratio of CD8 T-cells to Tregs. *(C)* Flow cytometric analysis of CD273 (PD-L2) expression on MDSCs. MEKi lead-in was associated with less CD273 expression on MDSCs. Histogram plot of CD273 intensity (*x* axis). MDSC, myeloid-derived suppressor cell; MEKi, MEK inhibition; PD-L2, programmed death ligand 2; Treg, T regulatory cell.

**Pharmacodynamic Analysis in Peripheral Blood Using Flow Cytometry.** The results comparing baseline versus on-treatment samples reported that arm A, patients who received trametinib in cycle 1 before the addition of pembrolizumab in cycle 2, had marked decreases in the levels of circulating myeloid-derived suppressor cells (MDSCs) (*p* = 0.05) and Tregs (*p* = 0.0018) relative to patients in arm B who received pembrolizumab first. (Fig. 3) The ratio of CD8+ T-cells to Tregs was also dramatically increased (*p* = 0.0011), supporting a favorable immunomodulatory effect of treatment (Fig. 3*B*).

**Figure 4:**
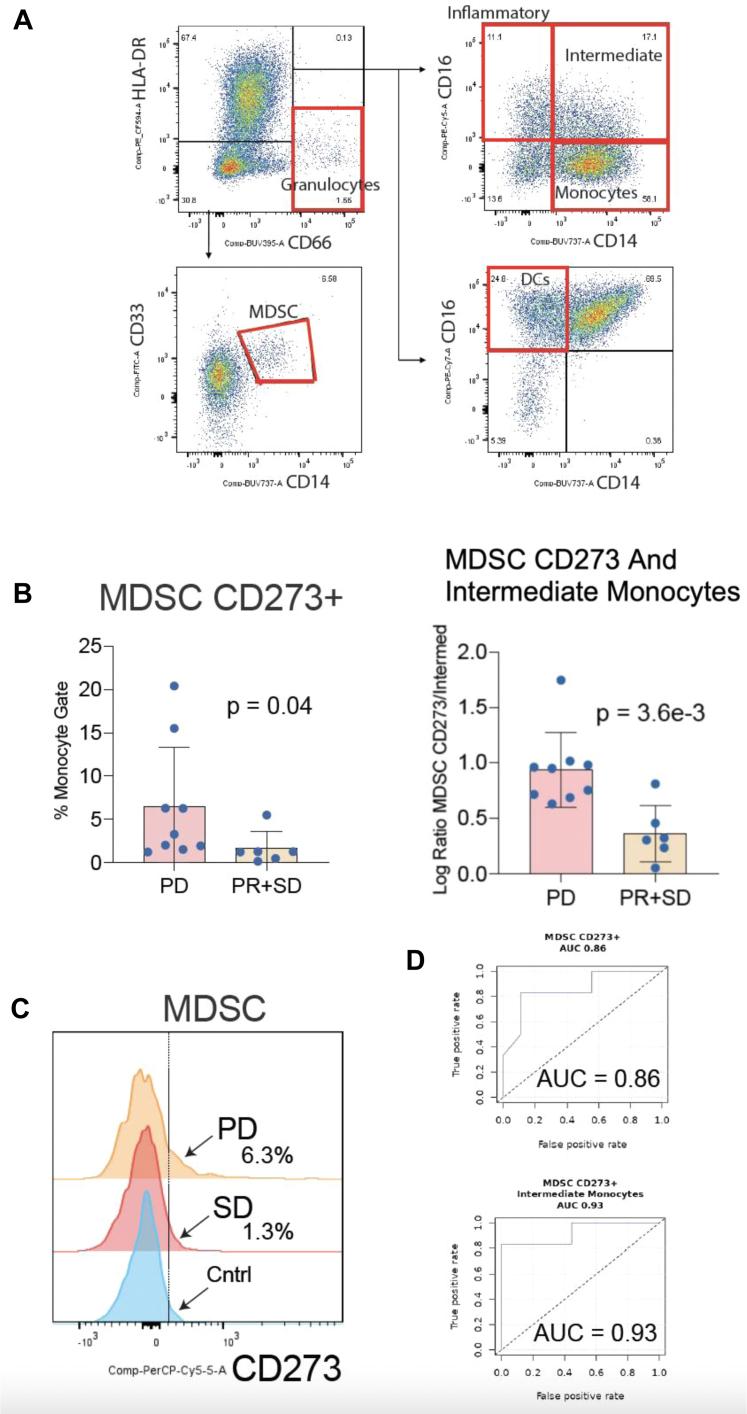
MDSCs are predictors of antitumor activity. *(A)* Gating strategy to identify MDSCs, inflammatory, intermediate, and classical monocytes. *(B*) Bar graph representing the frequency of CD273+ MDSCs at baseline in patients who would develop PD on therapy compared with those who would experience SD or a PR to therapy. *(C)* Flow cytometric analysis of CD273 (PD-L2) expression on MDSCs. PD was associated with increased CD273 expression on MDSCs. Histogram plot of CD273 intensity (*x* axis). *(D)* ROC curves representing the utility of CD273+ MDSC frequency and CD273+ MDSC to intermediate monocyte ratio as a predictor of de novo PD in patients with NSCLC treated with pembrolizumab and trametinib combination therapy. MDSC, myeloid-derived suppressor cell; PD, progressive disease; PD-L2, programmed death-ligand 2; PR, partial response; ROC, receiver operator characteristic; SD, stable disease.

**Circulating MDSCs and Antitumor Activity (SD + PR).** To explore immunophenotypes that could predict response to therapy, flow cytometry data from baseline samples was parsed into two groups to compare patients who experienced PD with those who experienced either disease (SD) or a PR. Results revealed that patients with a low percentage of programmed death-ligand 2 (PD-L2, aka CD273)–expressing MDSCs in pretreatment samples were more likely to respond to therapy (*p* = 0.04; Fig. 4*A*, *B*). To evaluate the ability of MDSCs as a biomarker of PD, a ROC was constructed and the area under the ROC (AUC) was calculated. This analysis revealed that the percentage of PD-L2-expressing MDSCs could prognosticate response to therapy with prominent accuracy, that is, separate patients who would benefit from the combination treatment (SD + PR) from those who would develop PD on therapy (AUC = 0.86; Fig. 4). The ability to distinguish SD plus PR patients from PD nonresponders at baseline was improved further by using the ratio of PD-L2-expressing MDSCs to intermediate monocytes (AUC = 0.93; Fig. 4*D*).

## Discussion

This phase 1 trial evaluated a combination of trametinib and pembrolizumab in patients with advanced NSCLC. The RP2D dose was determined to be 2 mg trametinib orally on days 1 to 10 and 200 mg pembrolizumab intravenous every 21 days, with lead-in trametinib for the first cycle (arm A). Toxicity was comparable with previously published studies with MEKi and PD(L)1 inhibitors in other solid tumors and was higher than pembrolizumab as a single agent.^18^ Overall, our study was negative, with limited clinical activity in KRAS and non-KRAS mutant NSCLC, in which this combination does not warrant further development in NSCLC.

The study was enriched for *KRAS*-mutated NSCLC with 87% of patients (13 of 15) having these alterations. This study was closed after the determination of the recommended expansion dose owing to the modest activity of the regimen, the changing landscape of treatment with chemotherapy plus ICI becoming standard first-line therapy, and the development of inhibitors targeting RAS-mutated lung cancers, particularly *KRAS* G12C, which is the most frequent KRAS amino acid substitution in NSCLC.

Despite the combination's limited overall activity, a PR with a PFS longer than 6 months was noted in a patient with a *KRAS* G12C mutant NSCLC who had de novo progression on nivolumab. Four patients (27%) had a PFS longer than 6 months, with three of these patients having tumors that progressed on prior ICI.

Numerically higher CD4+ and CD8+ T-cells and significantly higher CD20+ B-cells in the tumor immune microenvironment at baseline support the local immunostimulatory effect of the treatment and were associated with improved clinical outcomes to trametinib and pembrolizumab (Fig. 2*A*, *B*, *C*, *D*). Increased levels of CD4+ and CD8+ T-cells and B-cells associated with improved clinical outcomes have also been observed in other ICI clinical studies.^19^ To identify possible mechanisms of how MEKi lead-in (i.e., trametinib given first as a single agent followed by checkpoint blockade) might improve immunotherapy efficacy, we performed high-parameter flow cytometry to characterize peripheral immune cell alterations associated with this regimen. Our results indicate that patients who received lead-in trametinib (arm A) experienced potentially beneficial alterations in circulating immune populations. Specifically, trametinib lead-in was associated with a decrease in both Treg and MDSC cell frequencies (Fig. 3). Tregs are a subpopulation of immunosuppressive T-cells that inhibit the function of effector T-cells and other adaptive immune cells, which can diminish the effectiveness of immune checkpoint blockade.^12^ MDSCs include immature monocytes or granulocytes that can directly suppress T-cell responses and are likely key contributors to a tumor-promoting microenvironment.^25^ Recent data has also shown that MDSCs can contribute to ICI failure.^26^ Thus, the ability of lead-in trametinib to reduce both Tregs and MDSCs could be beneficial to patients receiving ICIs. A benefit of MEKi lead-in was also supported by the finding that arm A had a dramatic increase in the ratio of CD8+ T-cells to Tregs, that is, a reduction in regulatory cells was associated with more effector T-cells. We speculate that MEKi lead-in has a favorable immunomodulatory effect and could improve responsiveness to ICI therapy. Given the small sample size, *p* values should be interpreted with caution. The uncertainty associated with variation within our sample, and the small sample size still leaves open the need for future investigation of how generalizable this might be to a larger sample.

Though our study was small, our study provides some clinical evidence that sequencing lead-in RAS–RAF–MAPK pathway inhibition before combining with ICI may be preferred. In Parts 4 and 5 of the Keynote-022 study that combined MEKi with trametinib with the PD-1 antibody pembrolizumab in solid tumors, higher response rates were noted with lead-in, intermittent dosing compared with concurrent continuous dosing of trametinib.^18^ In melanoma, the IMspire150 trial of lead-in BRAF and MEKi with vemurafenib and cobimetinib combined with PD-L1 blockade with atezolizumab met its primary end point of improved PFS.^27^ Nevertheless, The COMBI-i trial of dabrafenib and trametinib with the PD-1 blocking antibody spartalizumab that did not employ lead in MEKi did not meet its primary clinical end point of PFS.^28^

Given the suboptimal response rates of NSCLC to immune checkpoint blockade, it is important to identify predictive biomarkers capable of identifying patients most likely to respond to therapy. To this end, we assessed the ability of peripheral immune cell frequencies to predict response to the pembrolizumab and trametinib combination immunotherapy. Results reveal that patients with higher pretreatment levels of PD-L2–expressing MDSCs in their peripheral circulation were less likely to clinically benefit from the pembrolizumab trametinib combination (Fig. 4*A*, *B*, *C*). Expanding this finding further revealed that the ratio of PD-L2-expressing MDSCs to intermediate monocytes at baseline was even a stronger indicator of clinical activity (PR + SD) (*p* = 0.0036, AUC = 0.93). PD-L2 is a T-cell inhibitory molecule expressed by MDSCs not blocked by pembrolizumab (Fig. 4*D*). Thus, it is not surprising that the higher the frequency of PD-L2-expressing MDSCs the more likely the patients would be unresponsive to pembrolizumab. Intermediate monocytes are CD14 (high) and CD16 (dim) cells that are thought to be important migratory cells, skilled at transendothelial migration. Unlike MDSCs, intermediate monocytes are proinflammatory and are skilled at antigen processing and presentation to T-cells. Nevertheless, our results cannot rule out a negative prognostic effect of the PD-L2-expressing MDSC population in identifying patients with unfavorable biology that would derive less benefit from most treatment regimens.

The combination assessed here performed adequately from a safety perspective and was comparable with similar MEKi plus PD(L)1 studies, with one DLT observed (G3 esophagitis) that was a known adverse event from trametinib. G3 investigation-related adverse events included pneumonitis, respiratory anemia, esophagitis, diarrhea, and retinal detachment. The patient who had a G3 pneumonitis event eventually developed grade 5 respiratory failure on comfort care in the setting of acute pulmonary emboli and multimicrobial bacterial and viral (coronavirus) pneumonia. In Parts 4 and 5 of the Keynote-022 trial that evaluated 42 solid tumor patients with trametinib and pembrolizumab a G3 pneumonitis event also occurred.^18^ Overall, there was more toxicity than what would be expected with anti-PD(L)1 therapy as a single agent.

The study included patients treated with prior PD(L)1 and PD(L)1 inhibitor naive patients. Two patients (2 of 15, 13%) had a response to treatment (one PD[L]1 treatment naive and one with prior treatment with nivolumab). Notably, most patients treated in the study had lung cancers that were PD-L1 immunohistochemistry–negative (10 of 15, 66%), which is predictive of a lack of benefit from ICI monotherapy. Given the changing landscape of advanced NSCLC treatment with first-line chemo-immunotherapy becoming standard of care and the approval of KRAS G12C direct inhibitors for that subset of *KRAS*-mutated NSCLC, the limited clinical activity and the toxicity observed, this study was closed and did not accrue to the dose expansion cohorts.

Overall, immune checkpoint blockade demonstrates heterogenous activity in *KRAS*-mutated NSCLC on the basis of KRAS amino acid substitution, smoking exposure, tumor mutational burden, and co-mutation status (i.e., STK11 or KEAP1).^29^ Thus, there is a large unmet need to optimize immunotherapy strategies in KRAS-mutant lung cancer to improve patient outcomes. Despite the limited clinical activity of trametinib and pembrolizumab, we were able to reveal the feasibility of baseline tissue analysis by quantitative immunofluorescence, serial blood sampling, and peripheral blood flow cytometry to detect immune cell alterations in the tumor microenvironment and in circulation that may underlie more favorable immunomodulatory activity, particularly with lead-in sequencing of MEKi therapy followed by MEKi plus PD(L)1 inhibition.^19^

There is emerging preclinical and clinical data on the clinical activity of KRAS G12C inhibitors combined with PD(L)1 inhibitors, though there are toxicity concerns, particularly hepatotoxicity with ICI and sotorasib.^30^ Notably, lead-in strategies of the KRAS G12Ci sotorasib attenuated side effects with the combination lead-with fewer overall and G3/4 TRAEs, discontinuations, and hepatotoxicity versus concurrent therapy (lead-in treatment strategies resulted in fewer overall and G3/4 TRAEs, discontinuations, and hepatotoxicity versus concurrent therapy).^31^ Pan-RAS inhibitors and KRAS direct inhibitors against non-G12C amino acid substitutions are also in clinical development and with promising activity, although their potential for ICI combination remains uncertain. Our study suggests that future studies combining ICI and direct RAS pathway inhibitors in clinical development should take into account sequencing and lead-in dosing schedules to optimize immune activation and antitumor activity.

## CRediT Authorship Contribution Statement

**Jonathan W. Riess:** Conceptualization, Data curation, Formal analysis, Funding acquisition, Investigation, Methodology, Project administration, Resources, Supervision, Validation, Writing - original draft, Writing - review & editing, Approval of the final version.

**Matthew S. Lara:** Formal analysis, Investigation, Methodology, Project administration, Writing - original draft, Writing - review & editing, Approval of the final version.

**Miguel Lopez de Rodas:** Data curation, Formal analysis, Investigation, Methodology, Project administration, Writing - original draft, Writing - review & editing, Approval of the final version.

**Guillaume Luxardi:** Data curation, Formal analysis, Investigation, Methodology, Writing - review & editing, Approval of the final version.

**Michiko Shimoda:** Data curation, Formal analysis, Investigation, Methodology, Writing - review & editing, Approval of the final version.

**Karen Kelly:** Data curation, Writing - review & editing, Approval of the final version.

**Primo N. Lara:** Data curation, Writing - review & editing, Approval of the final version

**Laurel Beckett:** Formal analysis, Investigation, Methodology, Writing - review & editing, Approval of the final version.

**Arta Monjazeb:** Methodology, Writing - review & editing, Approval of the final version.

**Kurt Schalper:** Data curation, Formal analysis, Investigation, Methodology, Writing - original draft, Writing - review & editing, Approval of the final version.

**Emanual Maverakis:** Data curation, Formal analysis, Investigation, Methodology, Writing - original draft, Writing - review & editing, Approval of the final version.

**David R. Gandara:** Conceptualization, Data curation, Methodology, Writing - review & editing, Approval of the final version.

## Disclosure

Dr. Riess has accepted personal fees for advisory boards/consulting from Boehringer Ingelheim, BMS, Roche-Genentech, Daiichi Sankyo, Regeneron, Janssen, Merus NV, Amgen, Catalyst, SeaGen/Pfizer, Bicycle Therapeutics, ArriVent and Oncohost; research support (To Institution) from ArriVent, Merck, Novartis, AstraZeneca, Spectrum, Summit, Revolution Medicines, IO Biotech, Nuvalent, Boehringer Ingelheim and travel fees from AstraZeneca and IO Biotech. Dr. Monjazeb has accepted personal fees for advisory boards/consulting from Merck, BMS, Genentech, Transgene, Incyte, Trisalus, IO Biotech. Dr. Gandara has accepted personal fees for advisory boards/consulting from Merck. All other authors declare no conflict of interest.

## Supplementary Data

Supplementary Tables1-3
