# Supplementary material for: A Phase 1/1B Trial of Pembrolizumab and Trametinib in Advanced NSCLC Enriched for KRAS Mutations
Source: JTO Clin Res Rep. 2025 Feb 12;6(6):100806. doi: 10.1016/j.jtocrr.2025.100806 (PMC12145753; doi:10.1016/j.jtocrr.2025.100806)
Supplement: Supplementary Tables1-3 [file mmc1.pptx]

## Slide 1
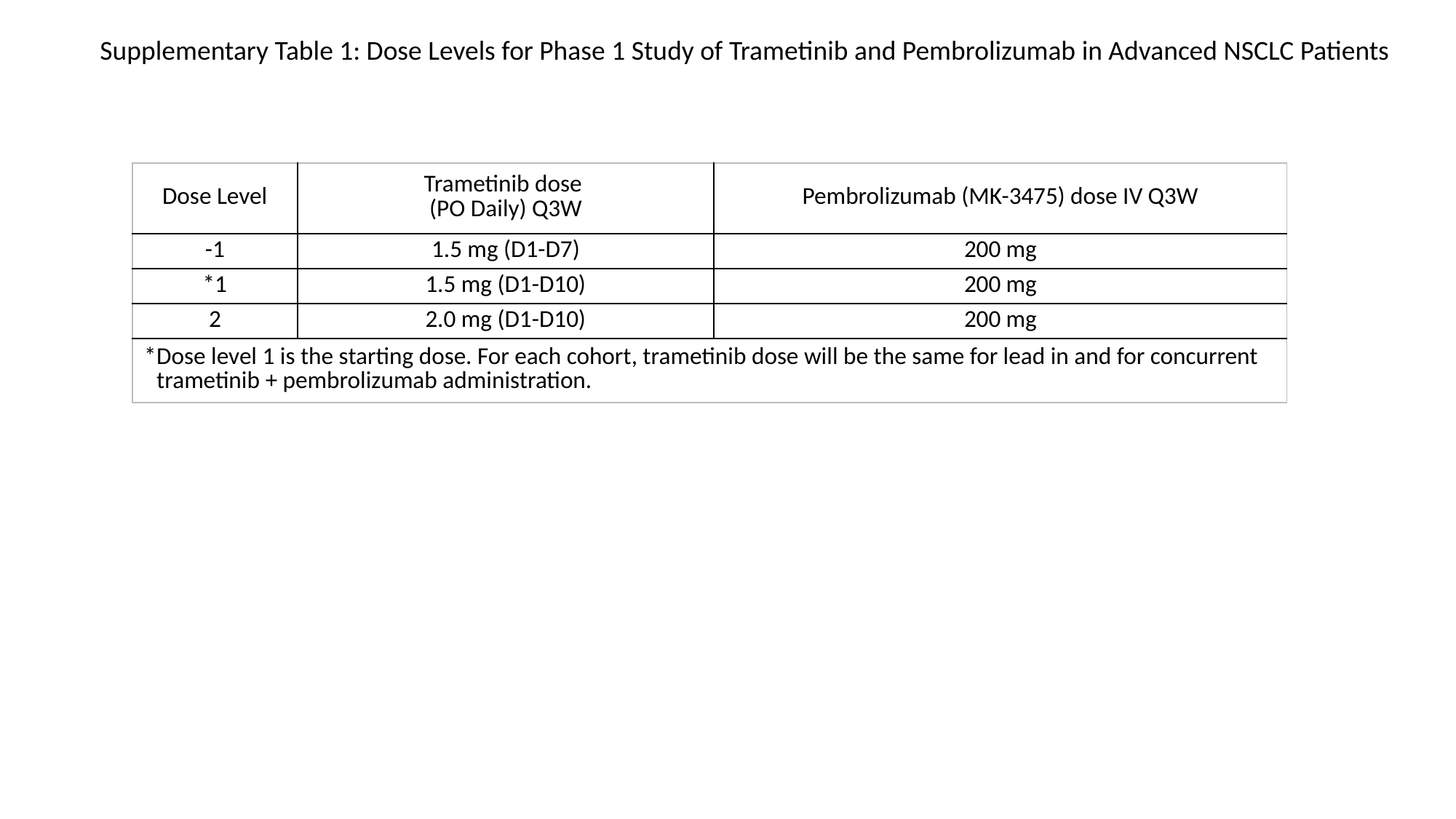

Supplementary Table 1: Dose Levels for Phase 1 Study of Trametinib and Pembrolizumab in Advanced NSCLC Patients
| Dose Level | Trametinib dose (PO Daily) Q3W | Pembrolizumab (MK-3475) dose IV Q3W |
| --- | --- | --- |
| -1 | 1.5 mg (D1-D7) | 200 mg |
| \*1 | 1.5 mg (D1-D10) | 200 mg |
| 2 | 2.0 mg (D1-D10) | 200 mg |
| \* Dose level 1 is the starting dose. For each cohort, trametinib dose will be the same for lead in and for concurrent trametinib + pembrolizumab administration. | | |

## Slide 2
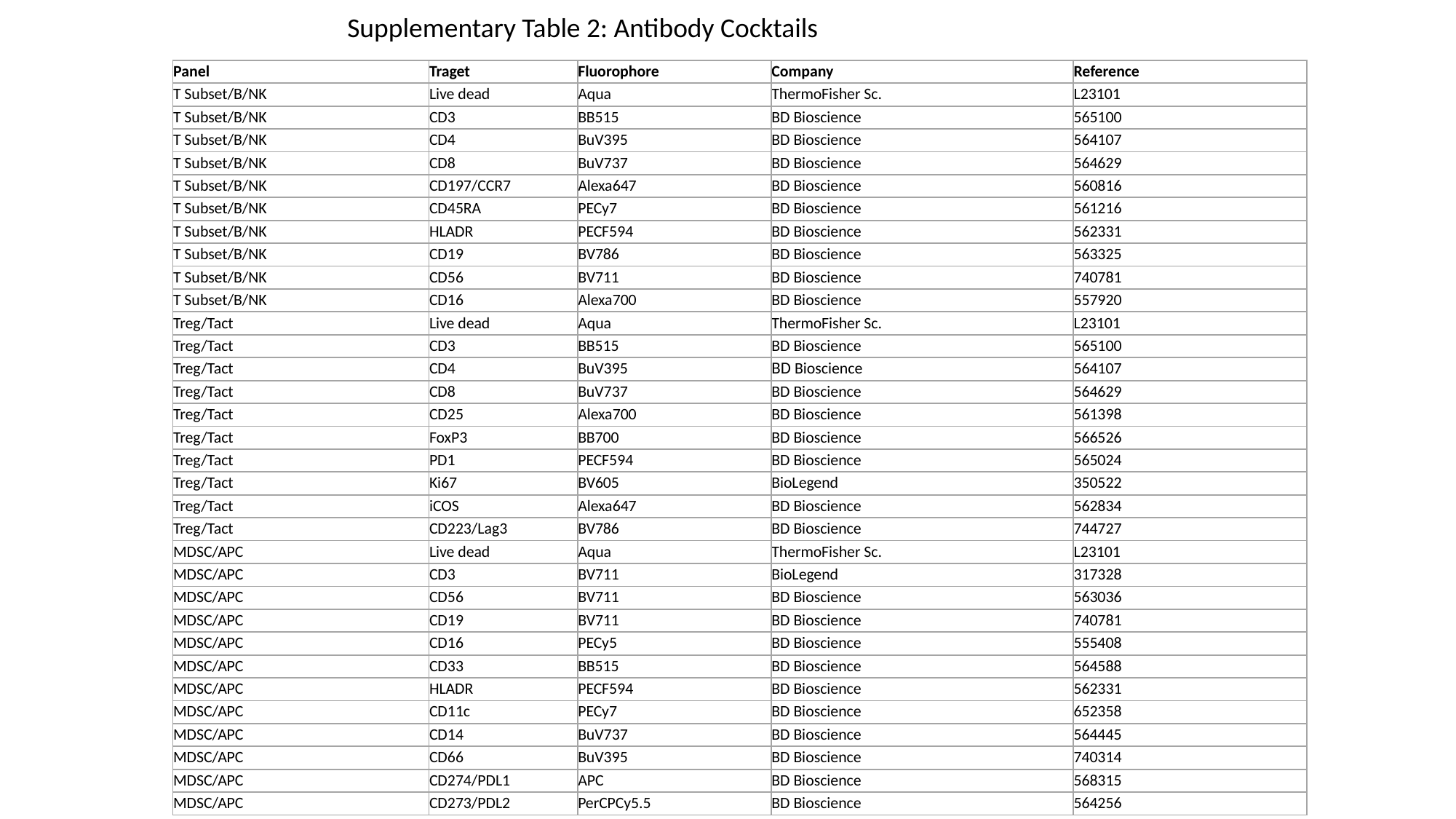

Supplementary Table 2: Antibody Cocktails
| Panel | Traget | Fluorophore | Company | Reference |
| --- | --- | --- | --- | --- |
| T Subset/B/NK | Live dead | Aqua | ThermoFisher Sc. | L23101 |
| T Subset/B/NK | CD3 | BB515 | BD Bioscience | 565100 |
| T Subset/B/NK | CD4 | BuV395 | BD Bioscience | 564107 |
| T Subset/B/NK | CD8 | BuV737 | BD Bioscience | 564629 |
| T Subset/B/NK | CD197/CCR7 | Alexa647 | BD Bioscience | 560816 |
| T Subset/B/NK | CD45RA | PECy7 | BD Bioscience | 561216 |
| T Subset/B/NK | HLADR | PECF594 | BD Bioscience | 562331 |
| T Subset/B/NK | CD19 | BV786 | BD Bioscience | 563325 |
| T Subset/B/NK | CD56 | BV711 | BD Bioscience | 740781 |
| T Subset/B/NK | CD16 | Alexa700 | BD Bioscience | 557920 |
| Treg/Tact | Live dead | Aqua | ThermoFisher Sc. | L23101 |
| Treg/Tact | CD3 | BB515 | BD Bioscience | 565100 |
| Treg/Tact | CD4 | BuV395 | BD Bioscience | 564107 |
| Treg/Tact | CD8 | BuV737 | BD Bioscience | 564629 |
| Treg/Tact | CD25 | Alexa700 | BD Bioscience | 561398 |
| Treg/Tact | FoxP3 | BB700 | BD Bioscience | 566526 |
| Treg/Tact | PD1 | PECF594 | BD Bioscience | 565024 |
| Treg/Tact | Ki67 | BV605 | BioLegend | 350522 |
| Treg/Tact | iCOS | Alexa647 | BD Bioscience | 562834 |
| Treg/Tact | CD223/Lag3 | BV786 | BD Bioscience | 744727 |
| MDSC/APC | Live dead | Aqua | ThermoFisher Sc. | L23101 |
| MDSC/APC | CD3 | BV711 | BioLegend | 317328 |
| MDSC/APC | CD56 | BV711 | BD Bioscience | 563036 |
| MDSC/APC | CD19 | BV711 | BD Bioscience | 740781 |
| MDSC/APC | CD16 | PECy5 | BD Bioscience | 555408 |
| MDSC/APC | CD33 | BB515 | BD Bioscience | 564588 |
| MDSC/APC | HLADR | PECF594 | BD Bioscience | 562331 |
| MDSC/APC | CD11c | PECy7 | BD Bioscience | 652358 |
| MDSC/APC | CD14 | BuV737 | BD Bioscience | 564445 |
| MDSC/APC | CD66 | BuV395 | BD Bioscience | 740314 |
| MDSC/APC | CD274/PDL1 | APC | BD Bioscience | 568315 |
| MDSC/APC | CD273/PDL2 | PerCPCy5.5 | BD Bioscience | 564256 |

## Slide 3
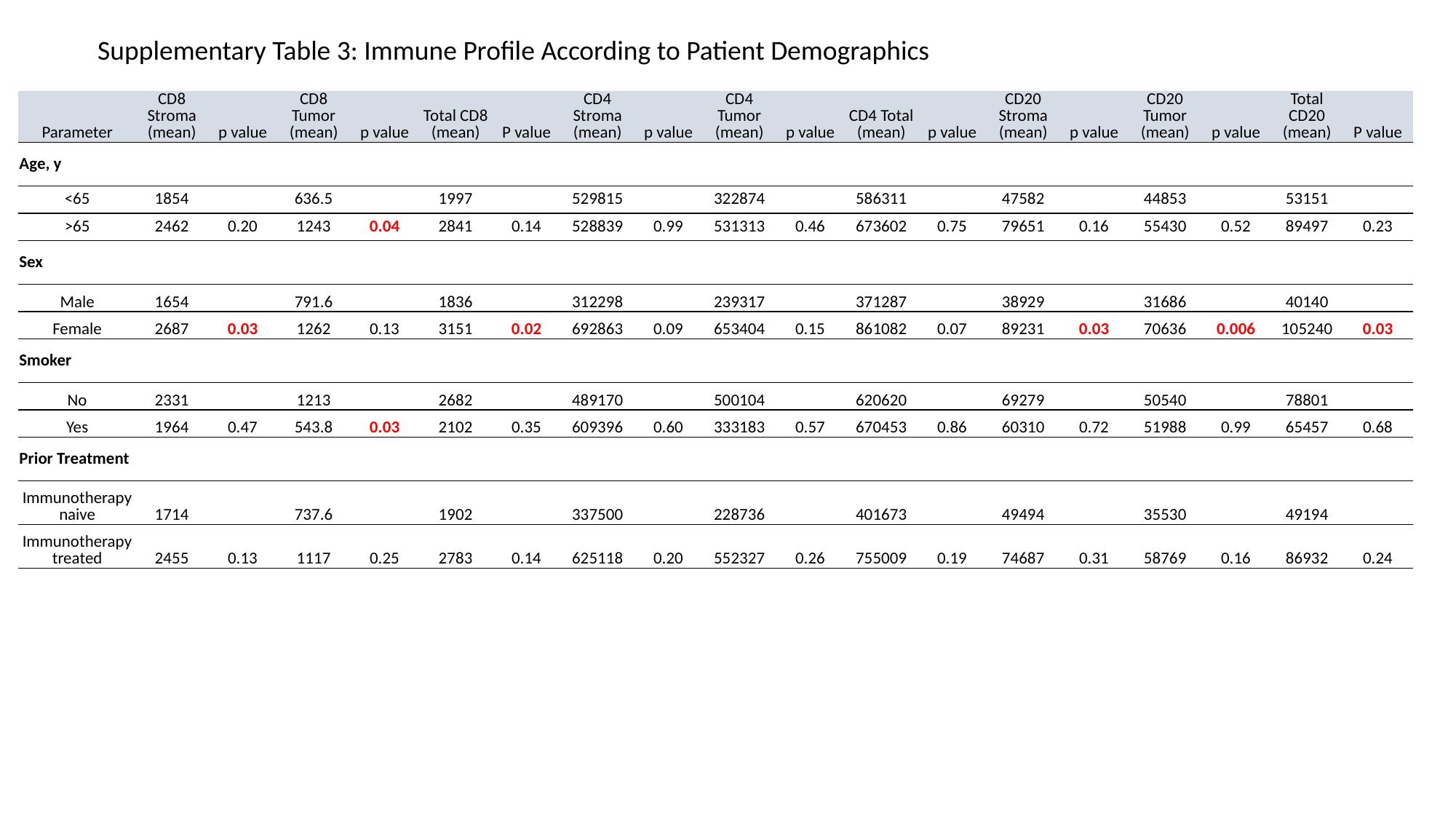

Supplementary Table 3: Immune Profile According to Patient Demographics
| Parameter | CD8 Stroma (mean) | p value | CD8 Tumor (mean) | p value | Total CD8 (mean) | P value | CD4 Stroma (mean) | p value | CD4 Tumor (mean) | p value | CD4 Total (mean) | p value | CD20 Stroma (mean) | p value | CD20 Tumor (mean) | p value | Total CD20 (mean) | P value |
| --- | --- | --- | --- | --- | --- | --- | --- | --- | --- | --- | --- | --- | --- | --- | --- | --- | --- | --- |
| Age, y | | | | | | | | | | | | | | | | | | |
| <65 | 1854 | | 636.5 | | 1997 | | 529815 | | 322874 | | 586311 | | 47582 | | 44853 | | 53151 | |
| >65 | 2462 | 0.20 | 1243 | 0.04 | 2841 | 0.14 | 528839 | 0.99 | 531313 | 0.46 | 673602 | 0.75 | 79651 | 0.16 | 55430 | 0.52 | 89497 | 0.23 |
| Sex | | | | | | | | | | | | | | | | | | |
| Male | 1654 | | 791.6 | | 1836 | | 312298 | | 239317 | | 371287 | | 38929 | | 31686 | | 40140 | |
| Female | 2687 | 0.03 | 1262 | 0.13 | 3151 | 0.02 | 692863 | 0.09 | 653404 | 0.15 | 861082 | 0.07 | 89231 | 0.03 | 70636 | 0.006 | 105240 | 0.03 |
| Smoker | | | | | | | | | | | | | | | | | | |
| No | 2331 | | 1213 | | 2682 | | 489170 | | 500104 | | 620620 | | 69279 | | 50540 | | 78801 | |
| Yes | 1964 | 0.47 | 543.8 | 0.03 | 2102 | 0.35 | 609396 | 0.60 | 333183 | 0.57 | 670453 | 0.86 | 60310 | 0.72 | 51988 | 0.99 | 65457 | 0.68 |
| Prior Treatment | | | | | | | | | | | | | | | | | | |
| Immunotherapy naive | 1714 | | 737.6 | | 1902 | | 337500 | | 228736 | | 401673 | | 49494 | | 35530 | | 49194 | |
| Immunotherapy treated | 2455 | 0.13 | 1117 | 0.25 | 2783 | 0.14 | 625118 | 0.20 | 552327 | 0.26 | 755009 | 0.19 | 74687 | 0.31 | 58769 | 0.16 | 86932 | 0.24 |
