# Supplementary material for: A Phase 1/1B Trial of Pembrolizumab and Trametinib in Advanced NSCLC Enriched for KRAS Mutations
Source: JTO Clin Res Rep. 2025 Feb 12;6(6):100806. doi: 10.1016/j.jtocrr.2025.100806 (PMC12145753; doi:10.1016/j.jtocrr.2025.100806)
Supplement: Supplementary Figures 1-2 [file mmc2.pptx]

## Slide 1
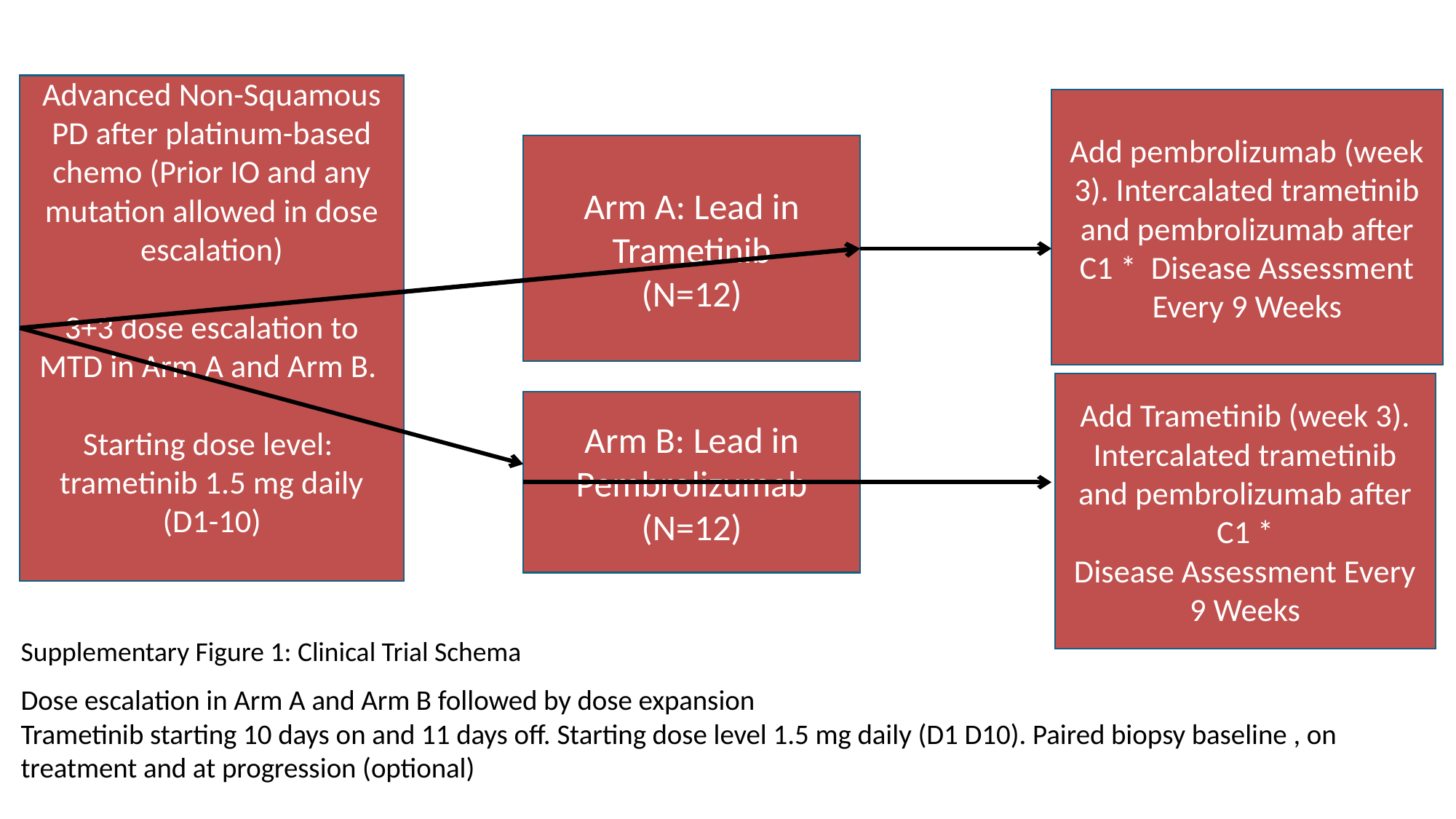

Advanced Non-Squamous
PD after platinum-based chemo (Prior IO and any mutation allowed in dose escalation)
3+3 dose escalation to MTD in Arm A and Arm B.
Starting dose level: trametinib 1.5 mg daily (D1-10)
Add pembrolizumab (week 3). Intercalated trametinib and pembrolizumab after C1 * Disease Assessment Every 9 Weeks
Arm A: Lead in Trametinib
(N=12)
Add Trametinib (week 3). Intercalated trametinib and pembrolizumab after C1 *
Disease Assessment Every 9 Weeks
Arm B: Lead in Pembrolizumab
(N=12)
Supplementary Figure 1: Clinical Trial Schema
Dose escalation in Arm A and Arm B followed by dose expansion
Trametinib starting 10 days on and 11 days off. Starting dose level 1.5 mg daily (D1 D10). Paired biopsy baseline , on treatment and at progression (optional)

## Slide 2
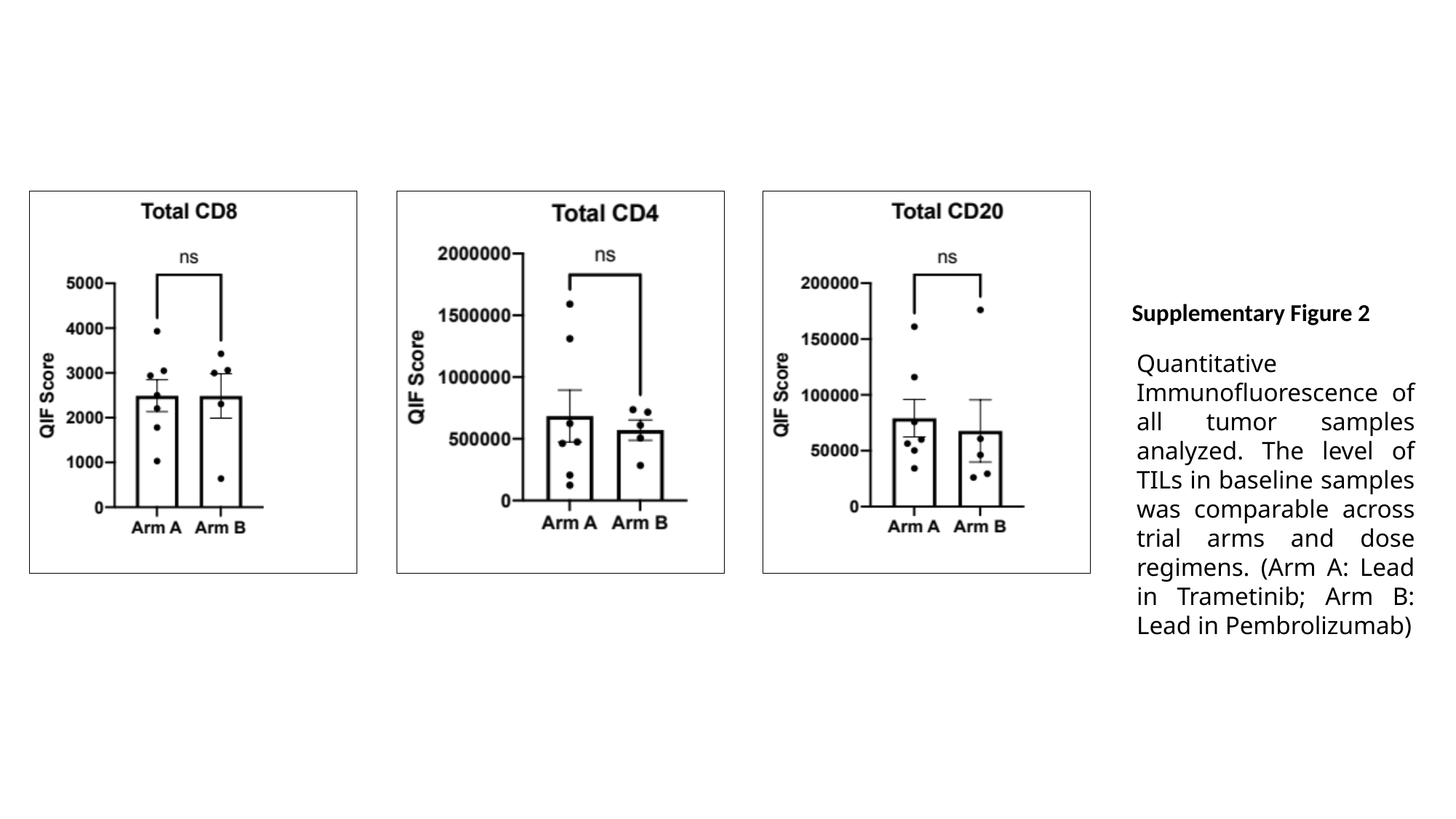

Supplementary Figure 2
Quantitative Immunofluorescence of all tumor samples analyzed. The level of TILs in baseline samples was comparable across trial arms and dose regimens. (Arm A: Lead in Trametinib; Arm B: Lead in Pembrolizumab)
